# Characterization of a novel bifunctional mutant of staphylokinase with platelet-targeted thrombolysis and antiplatelet aggregation activities

**DOI:** 10.1186/1471-2199-8-88

**Published:** 2007-10-07

**Authors:** Hongshan Chen, Wei Mo, Huabo Su, Yanling Zhang, Houyan Song

**Affiliations:** 1 The Key Laboratory of Molecular Medicine, Ministry of Education, Dong' an Road 130^#^, Fudan university, Shanghai, 200032, P. R. China; 2 Department of Biochemistry and Molecular Biology, Medical College, Qi xiu Road 19#, Nan Tong University, Nantong 226001, P. R. China

## Abstract

**Background:**

Although staphylokianse (SAK) is among the most promising blood dissolving agents, it is far from ideal. It is interesting to hypothesize that the clot lysis efficacy of SAK can be enhanced with direct active platelet binding ability, and at the same time the rethrombosis complication after successful recanalization can be minimized with an antiplatelet aggregation activity. The present study was performed to characterize the functional properties of RGD-SAK, a novel mutant of staphylokinase (SAK).

**Results:**

By using site-directed mutagenesis, an Arg-Gly-Asp (RGD) motif was engineered in the staphylokinase (SAK). This mutant of SAK designated RGD-SAK was expressed, purified and characterized. Biochemical analysis indicated that RGD-SAK maintained the similar structure and the fibrinolytic function of SAK. Measurement of platelet binding activity *in vitro *demonstrated that RGD-SAK had a much higher affinity with platelets than SAK. *In vitro *platelet-rich clot lysis assay demonstrated that the engineered mutant outperformed the non-manipulated SAK. The time required for 50% platelet-rich clot lysis and the concentration required to obtain 50% clot lysis (C_50_) were reduced significantly across different concentrations of RGD-SAK comparing with SAK. Meanwhile, RGD-SAK was found to inhibit ADP-induced platelet aggregation in a concentration-dependent manner while SAK had negligible effect on platelet aggregation.

**Conclusion:**

RGD-SAK possessed the bifunction to target platelet-rich clots and to block platelets aggregation, and thus may serve as a more potential thrombolytic agent with platelet-targeted thrombolytic and antiplatelet aggregation activities in compared with SAK.

## Background

Acute myocardial infarction is among the most prominent causes of death in the Western world. It is commonly caused by the formation of a pathologic clot that results in obstructing the blood flow to heart tissues. Staphylokinase (SAK), a 136-amino acid protein from certain lysogenic *Staphylococcus aureus *strains, is a plasminogen activator and a promising blood clot-dissolving agent with clinical potency that is at least as good as tPA [1,2]. SAK does not bind directly to fibrin, it can bind indirectly through the fibrin binding of plasmin(ogen) by forming a 1:1 stoichiometric SAK-plasmin(ogen) complex. The resulting SAK-plasmin complex can then function as the plasminogen activator to convert plasminogen to plasmin for clot lysis [3]. Although staphylokianse (SAK) is among the most promising blood dissolving agents, it is far from ideal. Recently, some studies have reported that, SAK could not mediate early reperfusion in 38% of treated patients and thrombolytic therapy employing staphylokinase is limited by rethrombosis of the very arteries being opened, which follows in a small but significant number of patients [1-3]. Development of more potent and faster-acting thrombolytic agents that can speed up the clot lysis process and have a lower rethrombosis rate would be desirable.

Now it is well known that platelets play a pivotal role in arterial thrombosis. Platelet-rich rather than fibrin-rich thrombosis was found to be responsible for many acute complications of angioplasty [4]. Meanwhile, studies indicated that the reformed secondary clots are usually platelet-rich after thrombolytic therapy [5]. Hence, it is interesting to hypothesize that the clot lysis efficacy of SAK can be enhanced by the application of the manipulated version of SAK that simultaneously targeted dissolve the platelet-rich clot and inhibit the reformation of blood clots by preventing platelets aggregation.

On activated platelets, the binding of surface glycoprotein GPIIb/IIIa to fibrinogen mediates platelet aggregation [6]. GPIIb/IIIa receptor antagonist, such as Arg-Gly-Asp (RGD) peptide had been proved to have obvious antiplatelet activity in thrombus formation experiments [7,8]. In order to improve the thrombolytic potential of SAK, as well as to introduce the antiplatelet activities, the mutant of SAK has been constructed. We substituted K^35 ^with Arg to constitute a RGD motif, resulting in a novel SAK variant, designated as RGD-SAK, which was supposed to be recognized by the activated GPIIb/IIIa on the surface of platelet membrane. However, it is not very clear whether the engineered mutant obtain the hypothesized effects. In this study, this mutant RGD-SAK was constructed and over-expressed in E coli. Biochemical properties, platelet-targeted thrombolysis, and antiplatelet aggregation of the purified target protein were determined.

## Results

### Construction, expression and purification of RGD-SAK

**Figure 1:**
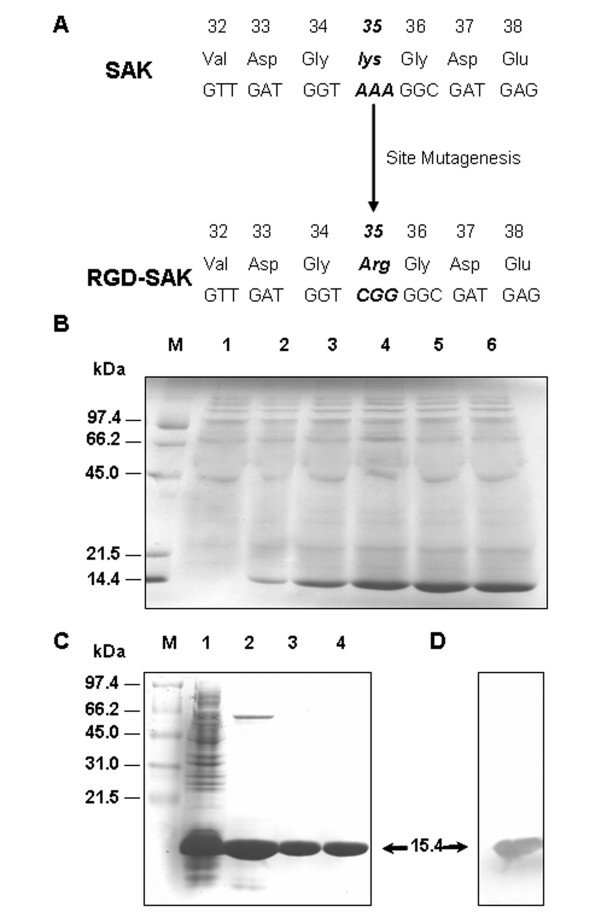
**Production of an engineered staphylokinase-based RGD-SAK**. (A) Amino acid residues and nucleotide sequences of SAK and RGD-SAK are shown. The lys^35 ^has been substituted with Arg to constitute a RGD motif and sequence analysis confirmed the successful mutagenesis. (B) Coomassie blue-stained 15% SDS-PAGE showing expression of RGD-SAK. Lane M, protein marker; lanes 1–6, induced for 0, 0.5, 1, 1.5, 2, 2.5 h. (C) Purification of recombinant protein RGD-SAK. Lane M, protein marker; lane 1, cytosol fractions; lane 2, purification after SP-Sepharose; lane 3, purification after Sephadex G-50; lane 4, purification after Q-Sepharose. (D) Western blot analysis with polyclonal anti-SAK antibodies.

Using PCR-mediated site-directed mutagenesis, the SAK variant RGD-SAK was constructed with Arg in place of K^35^, and the desired mutant was identified by DNA sequencing (Fig. 1A). After temperature induction, RGD-SAK was over-expressed due to the temperature inducible pL and pR promoter in the expression vector pLY-4. SDS-PAGE showed that the target protein was expressed after 0.5 h of induction and reached peak after 2.5 h (Fig. 1B). The protein was isolated by homogenization and purified by sequential chromatography through SP-Sepharose, Sephadex G-50, and Q-Sepharose (Fig. 1C). High purity protein was obtained (over 98% by densitometric scanning). Western blot (Fig. 1D) revealed that the RGD-SAK protein had a similar antigen binding capacity to SAK. Using known concentrations of pure SAK as the standard, the yield per liter culture of purified RGD-SAK was estimated to be 0.3 g and more than 60% was recovered in purification.

### Physicochemical and biological characterization of purified RGD-SAK

**Figure 2:**
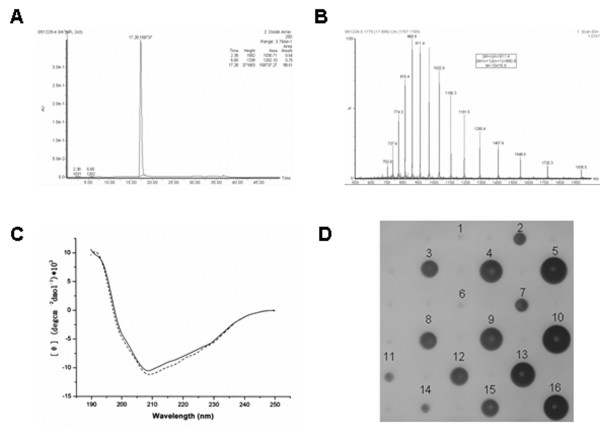
**Physicochemical and biological properties of RGD-SAK**. (A) HPLC analysis of RGD-SAK. A single symmetrical peak with a purity > 98% was indicated in the HPLC analytical chart. (B) Molecular mass determination of purified RGD-SAK by MALDF-TOF mass spectrometry. Peaks corresponding to various protonated RGD-SAK species are marked. The mass analysis determined that the RGD-SAK has a molecular weight of 15.4 KDa. (C) The secondary structure contents of RGD-SAK and SAK analyzed by far-UV CD spectrum. The secondary structure revealed by the curve of the two proteins were very similar. (D) RGD-SAK fibrinolysis activity assayed on fibrin plate. The numbers above the well represent various concentration of samples. 1–5 represent various concentrations of standard SAK (6.5 IU, 12.5 IU, 25 IU, 50 IU, 100 IU/well, respectively). Duplicate wells (6–10) corresponding to 1–5 were included. 11–13 represent various diluted RGD-SAK determined by comparing with the standard SAK. Three duplicated sets (14–16, 17–19, 20–22) of RGD-SAK corresponding to 11–13 were also shown.

To analyse the homogeneity of the puried RGD-SAK, RP-HPLC analysis was performed and purity analysis gave a single symmetrical peak with a purity > 98%. No significant peaks of impurities were observed at the RP-HPLC spectrum (Fig. 2A). Molecular mass spectrometric determination showed that the mono-protonated RGD-SAK molecule had a molecular weight of 15.4 KDa (Fig. 2B), which agreed very well with the apparent molecular mass (15.4 KDa).

To explore the structure of RGD-SAK, the purified sample was subjected to both N-terminal sequencing by the Edman degradation procedure and far-UV CD spectrum analysis. Sequencing of the first fifteen amino acid residues from the RGD-SAK (SSSFDKGKYKKGDDA) matched exactly with the mature SAK sequence. Analysis of the far-ultraviolet CD spectra (Fig. 2C) showed that SAK comprised 18.2% α-Helix, 36.7% β-Sheet, 45.0% turns and random coils while RGD-SAK comprised 19.3% α-Helix, 36.9% β-Sheet, 43.9% turns and random coils. These results revealed that the secondary structure of the two proteins were very similar and suggested that the substitution of K^35^with Arg did not change the secondary structure of SAK significantly.

The produced RGD-SAK was evaluated for safety and fibrinolytic potencies. The purified, sterilized preparations were found to contain low endoxin levels (< 3 EU/mg), which accorded with the clinical injection grade, and were devoid of acute toxicity in mice after intravenous bolus injection at a dose of 3 mg per mice in groups of 5 mice. Fibrinolytic activity of RGD-SAK was also determined on the fibrin plate (Fig. 2D). Comparing with the standard SAK, the fibrinolytic activity of RGD-SAK was 120,000 IU/mg, which was at least equal to or slightly higher than SAK. The result revealed that the introduction of RGD sequence into SAK had not altered its fibrinolytic activity. The plasminogen activation rate (determined by k_cat_/*K*_m_, *K*_m _represents the Michaelis constant, k_cat _represents the catalytic rate constant) was also examined. In mixtures of plasminogen with an equimolar SAK, the active site, as monitored with the chromogenic substrate S-2251, is rapidly exposed. Using this formed SAK-plasmin complex, the kinetic parameters for the activation of plasminogen were determined. Lineweaver-Burk analysis of the hydrolysis of S-2251 by plasmin-SAK or plasmin-RGD-SAK indicated that the k_cat_/*K*_m _values are comparable (0.033, 0.037 respectively. Table 1), revealing that RGD-SAK has SAK-like plasminogen activation property.

**Table 1:** The kinetics of equimolar mixture of human plasmin with RGD-SAK

	*K*_m _(μM)	k_cat _(s^-1^)	k_cat_/*K*_m _(μM^-1^s^-1^)
SAK	0.39 ± 0.03	0.015 ± 0.002	0.033 ± 0.003
RGD-SAK	0.78 ± 0.05	0.031 ± 0.001	0.037 ± 0.005

Data obtained from three experiments.

### Fibrin and platelet binding assay

**Figure 3:**
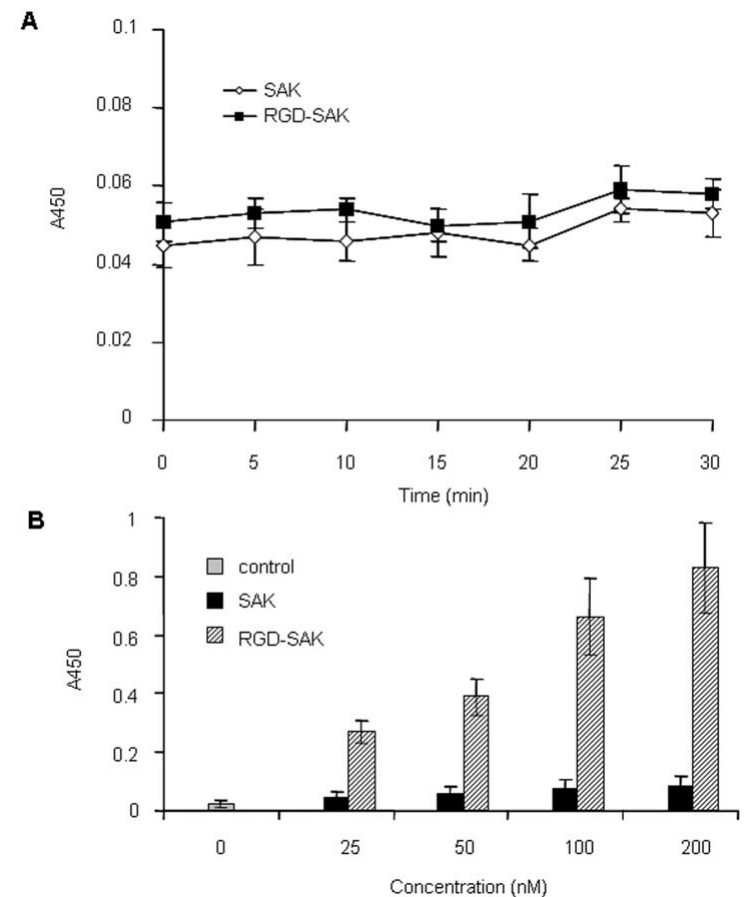
**Fibrin and platelet binding assay**. (A) The quality of RGD-SAK absorbed by fibrin was similar to that of SAK. Data represent means ± SD obtained form three independent experiments. (B) ELISA was taken to measure the OD450, which represented the quantity of SAK or RGD-SAK bound to the active platelet. The statistical differences are significant for each tested concentration (RGD-SAK vs SAK, *P *< 0.01). The means ± SD came from three separate experiments.

To assess the fibrin binding ability of RGD-SAK, ELISA experiments with immobilized fibrin were performed. The results (Fig. 3A) showed that a weak fibrin binding occurred to both SAK and RGD-SAK, which showed no significantly statistic difference at a final concentration of 200 nM. In contrast, to examine the possible platelet-targeted binding ability of RGD-SAK due to the presence of RGD sequence, the *in vitro *platelet binding assay was performed. Data showed that the platelet-targeted binding quantity of RGD-SAK was much higher than that of SAK at various examined concentrations (*P *< 0.01, Fig. 3B). ELISA for platelet binding also indicated that RGD-SAK could bind the active platelet in a dose dependent manner. As shown in the same figure, SAK almost had no platelet-binding activity.

### Fibrin clot and platelet-rich clot lysis assay

**Figure 4:**
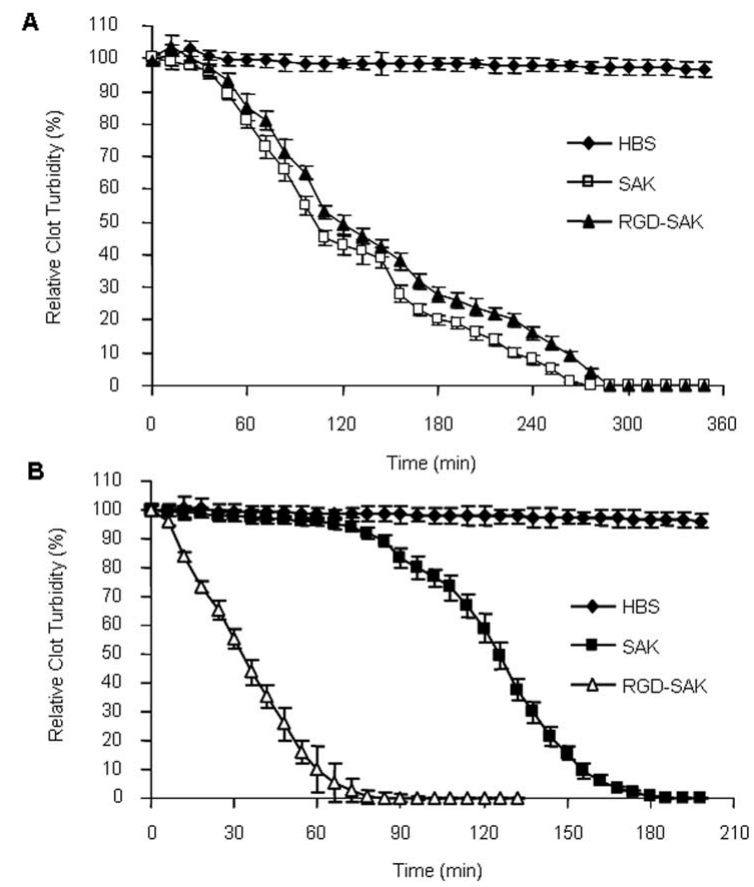
**Time course of fibrin clot and platelet-rich clot lysis assay**. The relative clot turbidity was calculated by detecting the decrease of the absorbance at OD405. The clots were either acted with HBS or with SAK or RGD-SAK. The experiments were repeated for three times. (A) *T*_50% _SAK and RGD-SAK calculated by the fibrin clot lysis assay were not significantly different at the final concentration 200 nM. (B) *T*_50% _was 118 ± 4.3 min for SAK and 57 ± 6.6 min for RGD-SAK calculated by the platelet-rich clot lysis assay at the final concentration 200 nM.

The efficiency of fibrin clot lysis was investigated on the ELISA plates by calculating the time required for 50% fibrin clot lysis (*T*_50%_). Data showed (Fig. 4A, Table 2) that the fibrinolytic efficiencies of the two proteins on the fibrin clot were not significantly different at various examined concentrations, consistent with their similar fibrinolysis activity assayed on fibrin plate. To examine whether the enhanced active platelet binding ability by RGD-SAK would lead to a faster clot lysis, we carried out platelet-rich clot lysis experiments which partially simulate the physiology situation. The time required for 50% platelet-rich clot lysis (*T*_50%_) by SAK (118 ± 3.4 min) was obviously longer (SAK vs RGD-SAK, *P *< 0.01) than that by RGD-SAK (57 ± 6.6 min) at the final concentration of 200 nM (Fig. 4B). The differences in *T*_50% _values between SAK and RGD-SAK were also dramatic (RGD-SAK vs SAK, *P *< 0.01) at the lower concentrations (50, 100 nm, table 2). The results in table 3 indicated that in comparison with SAK, the *T*_50% _of RGD-SAK was shortened by 1.75, 1.57 and 2.07 folds respectively at the corresponding concentrations (50, 100 and 200 nm). The degree of thrombolysis by different amounts of SAK and RGD-SAK was also determined graphically from plots of clot lysis versus the concentration of corresponding protein. Equieffective concentrations of test compound (C_50_, causing 50% fibrin clot lysis in 3 hours) determined from plots of clot lysis at 3 hours versus the concentration were 80 ± 8.5 nM for SAK, 76 ± 8.3 nM for RGD-SAK, which were not statistically different in this system (Table 2). 50% thrombolysis C_50 _in 2 hours required 200 ± 8.3 nM of SAK and 38 ± 3.9 nM of RGD-SAK, as determined by interpolation, thus the thrombolytic effect of RGD-SAK in this system was about 5.3 times shorter than that of SAK (Table 2).

**Table 2:** The time required to lyse 50% of fibrin clot or Platelet-rich clot (*T*_50%_) and the concentration required to obtain 50% clot lysis (C_50_) with SAK or RGD-SAK

Compound	Concentration (nM)	***T***_50% _of fibrin clot (min)	***T***_50% _of platelet-rich clot (min)	C_50 _of fibrin clot lysis (nM)	C_50 _of platelet-rich clot lysis (nM)
SAK	50	195 ± 8.7	198 ± 8.6	80 ± 8.5	200 ± 8.3
	100	161 ± 9.3	166 ± 7.8		
	200	138 ± 8.5	123 ± 5.6		
RGD-SAK	50	189 ± 9.9	93 ± 3.6	76 ± 8.3	38 ± 3.9
	100	151 ± 6.3	59 ± 2.9		
	200	131 ± 9.7	33 ± 3.8		

The activity of RGD-SAK or SAK was comparable in fibrin clot lysis assay at various corresponding concentrations.*T*_50% _was significantly shortened for each corresponding concentration (RGD-SAK vs SAK, *P *< 0.01). Results shown represent means ± SD from 6 clots for each concentration of SAK or RGD-SAK. C_50 _of fibrin clot lysis was not significantly different between SAK and RGD-SAK, while C_50 _of platelet-rich clot lysis caused by SAK was much higher than that of RGD-SAK (*P *< 0.01).

### Antiplatelet aggregation assay

**Figure 5:**
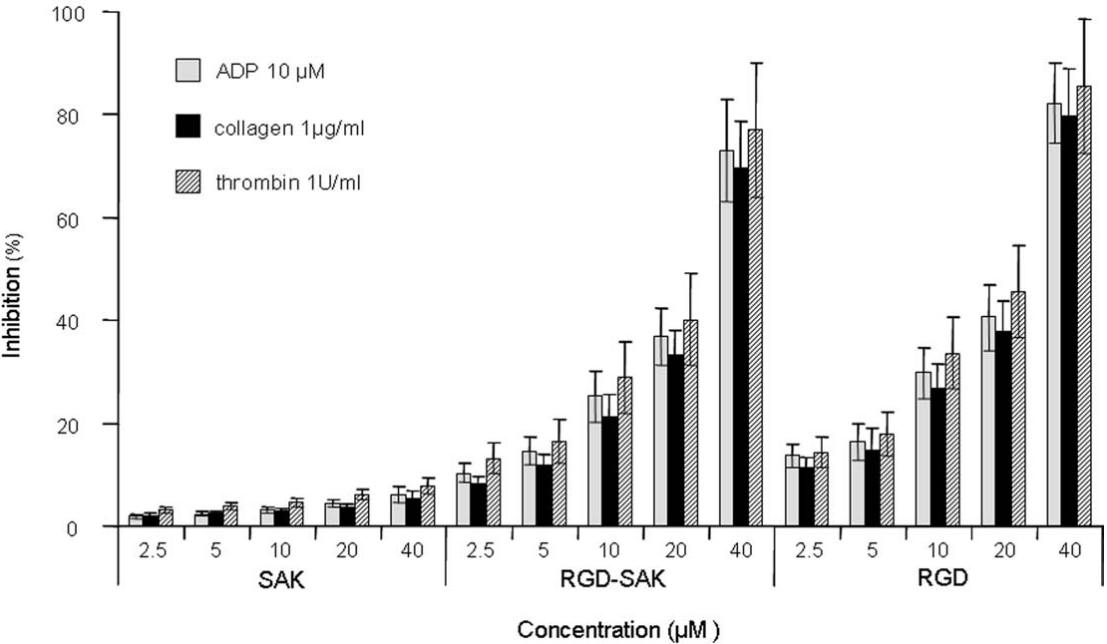
**The effects of SAK and RGD-SAK on platelet aggregation**. The means ± SD came from six independent experiments. Results are expressed as a percent inhibition in relation to platelets incubated with no recombinant proteins taken as 100%. The statistical differences between the inhibitory effects of RGD-SAK and SAK for each tested concentration are significant (*P *< 0.01).

It is predicted from the molecular design that the bifunctional RGD-SAK not only possesses the fibrinolytic activity, but may also inhibit the platelet aggregation. This was testified by the antiplatelet aggregation assay *in vitro *(Fig. 5). A significantly higher inhibitory effect of RGD-SAK on platelet aggregation stimulated by ADP (10 μM) or collagen (1 μg/ml) or thrombin (1 U/ml) was obtained compared with that of SAK, which had negligible effect on platelet aggregation. The dose dependent inhibition of human platelet aggregation by RGD-SAK was also indicated (Fig. 5). The inhibitory effect of RGD-SAK was comparable to RGD-peptide alone.

## Discussion

In this study, we report the construction and evaluation of a new class of bifunctional proteins consisting of two functional modules, RGD and SAK. To develop the novel SAK variant designated RGD-SAK with platelet-targeted thrombolytic and antiplatelet aggregation activity, K^35 ^in SAK was substituted with Arg to constitute a RGD motif, which was testified to be recognized by the activated GPIIb/IIIa. As three-dimensional structures of SAK and its ternary complex with μPG (micro-plasminogen) have been resolved [9], various attempts to manipulate and optimize the structure and function of SAK have been reported [3-5]. One of them is to construct a targeted thrombolytic agent. In some research, the Arg-Gly-Asp (RGD) peptide was constructed in the SAK due to its affinity with GPIIb/IIIa on the membrane of activated platelets. However, the anticipated targeting effect to platelet-rich clots was either partially or completely lost in some studies, likely attributed to the positioning of RGD, which should not be blocked by unfavorable spatial structure or be cleaved off during the plasminogen activation process [10,11]. By analysing the molecular modeling of the mutants, our previous study [12] suggested that loop 1 in SAK would be a rational introducing site for RGD. Research [13] found that the secondary structure sheet 1 (G22-D33) of SAK can be connected with sheet 2 (N37-I49) by loop 1, where the inserted RGD motif is supposed to be exposed on the solvent accessible surface of the SAK molecule and thus may allow access to the intergrin receptors. In addition, loop 1 is far from the active cleft in the SAK ternary complex [9], hence the mutation may have little effect on the SAK complex activity.

For biochemical and biological analysis of RGD-SAK, a convenient and effective method was developed to express and purify the target protein, which also provided an empirical basis for the establishment of standard operating procedure (SOP) for further large scale production. Compared with the conventional induction by IPTG (Isopropyl β-D-1-thiogalactopyranoside) [14], the temperature inducible expression under the control of pL and pR promoter in RGD-SAK expression is more feasible and cost-saving for fermentation. By optimizing fermentation condition, e.g. bacteria density and induction time, RGD-SAK was present in intracellular soluble state instead of inclusion body as reported previously [15], which is highly desirable for further purification. To obtain the highly purified and endotoxin-free protein, the cytosolic fractions after homogenization were subjected to cation chromatography and gel filtration to separate RGD-SAK, followed by anion chromatography to eliminate endoxin. The safety evaluation experiments testified that the highly purified, sterilized preparations contained low endotoxin levels, and were devoid of acute toxicity, which is safe and suitable for a more detailed clinical investigation.

Biochemical analysis indicated that RGD-SAK maintained the similar structure and the fibrinolytic function of SAK. The uniform N terminal sequence and similar CD spectrum suggested that these two molecules had similar structure. The measurement with fibrin plate and fibrin clot respectively revealed that the fibrinolytic activity of RGD-SAK was at least equal to that of SAK. The study further revealed that RGD-SAK can form a 1:1 RGD-SAK-plasmin complex which displayed an amidolytic activity comparable with that of the native SAK-plasmin binary complex. The similar catalytic efficiencies determined by k_cat_/*K*_m _of RGD-SAK in comparison with SAK revealed that the specific plasiminogen activating potential of the mutant remained almost intact. These above results indicated that the RGD existence in SAK would not disrupt the structure and the fibrinolytic function of SAK.

The platelet binding experiment indicated that RGD-SAK could bind the activated platelet in a dose dependent manner and with a higher affinity than SAK, which has weak ability to bind the platelet. In the platelet-rich clot lysis assay, the *T*_50% _values for RGD-SAK were significantly shortened. Analysis of these *T*_50% _values revealed a significant increase in platelet-rich clot lysis rate by RGD-SAK compared with SAK. Our data also demonstrated that RGD-SAK did not lead to enhanced fibrin clot lysis under *in vitro *conditions in comparison with SAK, which was correlated with their similar ability to bind the fibrin clots. By interpolation of the data of clot lysis assay, the C_50 _of the two proteins was similar in fibrin-rich clot while the C_50 _of RGD-SAK in platelet-rich clot was much shorter than equalmole SAK. These results suggested that RGD-SAK can target to the platelet-rich clots due to the direct selective binding to the activated platelets but not to fibrin which is also an important constituent of platelet-rich clots. Based on these results, we can conclude that RGD-SAK can accumulate on the platelet surface and thus enrich the thrombolytic activity at the site of the thrombus. Hence, it is possible that RGD-SAK would be directly targeted to thrombus *in vivo *because of their high affinities to platelets, which can therefore lyse the platelet-rich clots more efficiently than SAK due to the increased local concentration on the thrombus. In addition, it would be interesting to consider that the platelets themselves can suppress clot lysis by mechanical cross-linking, promotion of clot stability as well as release of fibrinolytic inhibitors [16]. Some recent *in vivo *studies [17,18] revealed that fibrinolysis can be accelerated by conjunctive use of anti-GP IIb/IIIa antibody. Hence, these findings imply that the RGD-SAK may accelerate lysis of platelet-rich clots via diverse mechanisms *in vivo*. Finally, it should be noted that a retarded renal clearance of RGD-SAK can also be expected due to its high affinities to the activated platelets and thus the resulting prolongated duration of action would allow use of low doses and/or less frequent administration of the agent. Therefore, if less RGD-SAK is required to achieve the same degree of clot lysis as SAK, the risk for side effects could also be minimized. We will further test the speculation by pharmacological and pharmacokinetic studies *in vivo*.

The use of RGD peptide as a platelet-targeted domain in the design of SAK mutant offers RGD-SAK another potential advantage of antiplatelet aggregation. It is well known that aggregation of blood platelets plays a pivotal role in the formation of thrombus and the RGD peptide is a component of ligands recognizing platelet intergrins [7,8]. Therefore, the protein containing RGD has the potential ability to inhibit platelet aggregation and thus to be used in the prevention of arterial rethrombosis. In the antiplatelet aggregation assay *in vitro*, we have shown that the addition of the RGD sequence to SAK resulted in acquisition of the ability to prevent platelet aggregation. In the present study, the RGD-SAK had obvious inhibitory effects on different inducers (ADP or collagen or thrombin)-induced platelet aggregation in a dose dependent manner, while the SAK had almost no effect on platelet aggregation. Meanwhile, the efficiency of antiplatelet aggregation by RGD-SAK was comparable with that of RGD sequence alone. For ADP, collagen and thrombin are the most powerful physiological inducers of platelet aggregation after arterial injury and during rethrombosis, so the present data indicated that RGD-SAK possessed the ability to block platelet aggregation and hence should be more effective than SAK in preventing the formation of rethrmobosis after successful thrombolytic therapy, which would also be worthy of further research *in vivo*. Noticeably, for the thrombolysis and rethrombosis *in vivo *is a complex process, thrombin was also found to be closely related to this action. So, it's very interesting to speculate that the addition of antithrombin functional element to SAK may also accelerate the successful lysis of the thrombus as well as the protection against rethrombosis. Recently such a protein has been synthesized by Szemraj *et al*. [19], which cued in us that fusion antithrombin element with RGD-SAK may form a even more potent and faster thrombolytic agent with anti-rethrombosis properties than SAK. We will further manipulate and screen the rational SAK mutant on the purpose in the future.

## Conclusion

In conclusion, the present study established the large scale expression and preparative purification method for RGD-SAK. The results of the *in vitro *study revealed that the purified mutant of SAK possessed the bifunction to target platelet-rich clots and to block platelets aggregation, and thus may be a more potent clot-dissolving agent for thrombolytic therapy compared with SAK.

## Methods

### Construction of E. coli expression vector of RGD-SAK

The DNA fragment encoding RGD-SAK was obtained by substituting K^35 ^with Arg to constitute a RGD motif using PCR-based mutagenesis on the template of pSTE-SAK constructed in our previous research [12]. in which gene encoding the mature region of SAK. The desired mutant was confirmed by DNA sequencing and cloned into expression vector pLY-4. Cytosolic expression was obtained by introducing the recombinant plasmids PLY-4-DGR into E coli JF1125 under the temperature inducible pL and pR promoter.

### Large scale expression and preparative purification of RGD-SAK

Two-step fermentations were used in the protein expression. Fourteen-liter M9CA medium was inoculated with 1 L of an overnight bacteria culture in LB medium in a 20 L fermentor (Bioengineering, Swiss), and was grown at 30°C for 5 h. The culture was then transferred into a 300 L fermentor with 200 L M9CA medium, and grew at 30°C for another 5 h. The soluble expression of RGD-SAK was induced at 42°C for 2.5 h before harvesting. For RGD-SAK purification, the cell pellet collected by centrifugation was homogenized with a high pressure homogenizer (APV-2000, Denmark). The supernatant obtained was purified by Chromatography system (AKTA Explore, GE) at 4°C on a 12.5 × 40 cm column of SP-Sepharose (Amersham-Pharmacia Biotech, USA), equilibrated with 0.02 M NaAc-HAc (pH 5.6), and eluted with a linear salt gradient (0.01–1 M NaAc-HAc-NaCl). RGD-SAK containing fractions were further purified by 12.5 × 100 cm Sephadex G-50 (Amersham-Pharmacia Biotech, USA) and concentrated by application on a 7.5 × 50 cm column of Q-Sepharose (Amersham-Pharmacia Biotech, USA) with stepwise elution (0.01–1.0 M PB-NaCl). The RGD-SAK containing fractions were pooled and adjusted to 1 mg/ml, and the material was sterilized by filtration through a 0.22 *μ*m Millipore filter. Purified recombinant protein was analyzed by 15% SDS-PAGE. Rabbit polyclonal anti-SAK antibodies were used for the detection of RGD-SAK by western blot analysis.

### Physicochemical characterization of RGD-SAK

The purity of this complex was confirmed by High-performance liquid chromatography (HPLC). The purified and concentrated samples were applied to a reversed phase C18 column (3.5 *μ*m, 2.1 × 100 mm) (Waters, Milford, MA, USA), using a 2690 Alliance HPLC (Waters, Milford, MA, USA). Samples were eluted with a 0.1% HCOOH (H_2_O): 0.1% HCOOH (CH_3_OH) gradient, 100:0 to 0:100 in 30 min at a flow rate of 0.2 ml/min. Protein mass spectrometry analysis was performed using Matrix-assisted Laser Desorption Ionization-Time of Flight (MALDITOF) Mass (Waters, Milford, MA, USA). Mass spectra of the samples were obtained using a micromass spectrometer equipped with electrospray ionization (ESI) source.

The structural analysis of the protein was performed using N-terminal sequencing and Circular dichroism spectroscopy (CD spectra). Briefly, Purified recombinant protein were transferred to a polyvinylidene difluoride (PVDF) membrane by electroblotting for N-terminal sequencing by automated Edman degradation procedure as described by Matsudaira [20]. The obtained sequence was compared with the cDNA-deduced polypeptide sequence. The secondary structure of the protein was estimated from spectral simulations based on reference CD spectra of Yang et al [21].

### Biological analysis of RGD-SAK

The methodology for evaluation of endotoxin contamination, bacterial sterility, and acute toxicity in mice of RGD-SAK was established according to Collen D et al. [22]. Purified preparations of RGD-SAK were planted on agar plates, which were then incubated under aerobic and anaerobic conditions, on McConkie and chocolate-agar media, and in thioglycolate and tryptic soy broth media to test bacterial sterility. The limulus amoebocyte lysate assay was used to determine the endotoxin contamination of the preparations according to the instructions of the manufacturer (sFDA). The acute toxicity of RGD-SAK preparations versus saline was evaluated by intravenous bolus injection of 200 *μ*l of the purified sterilized material at a dose of 3 mg per mice in 6 mice every group (All animals care and animal experimentation involved in this work were in accordance with NIH guidelines and the approved procedures of the Institutional Animal Use and Care Committee at Fudan University.). The animals were observed for living state and acute reactions (tachypnea, restlessness, stupor) during an 8-day observation period.

### Fibrinolytic activity assay on fibrin plate

Fibrinolytic activity of SAK and RGD-SAK was measured on fibrin plate [23]. Petri dishes containing 0.5% agrose, 0.055 IU/ml thrombin, 5.5 *μ*g/ml plasminogen (PG) and 0.52 *μ*g/ml fibrinogen were prepared. On this solidified fibrin plate, wells of equal diameter were punched and 10 *μ*l/well of diluted appropriate concentration of SAK (1000 IU/ml, SFDA) or RGD-SAK was added and kept at 37°C for 16 h. The diameter of the halo around the well was measured to calculate the functional activity of SAK or RGD-SAK.

### Activation of human plasminogen

The kinetics of plaminogen activation was determined according to Schlott B *et al*. [24]. Plasminogen was incubated with equimolar SAK or RGD-SAK at 37°C for 1 h to generate the SAK-plasmin or RGD-SAK-plasmin bimolecular complex. The performed activator complex (final concentration 10 nM) was then mixed with different concentration of plasminogen (final concentration 0.625 *μ*M, 1.25 *μ*M, 2.5 *μ*M, 5 *μ*M, 10 *μ*M) and generation of plasmin was continuously monitored at 405 nm with S-2251 (3 mM). The kinetics constants were determined from Lineweaver-Burk plots.

### Measurement of fibrin binding activity

Microtiter plate was coated overnight with cross-linked fibrin as described by Wu et al. [25]. Briefly, Fibrinogen (5 *μ*g/ml) was added into the wells and left overnight at 4°C, then blocked with 2 mg/ml BSA for 2 h. The fibrinogen was incubated with PBS containing human thrombin (1 NIH unit/ml) and CaCl_2 _(20 mM) for 2 h at 37°C to form cross-linked state. The fibrin was partially digested with plasmin at 1 pmol/well for 10 min at the room temperature, then the microplate was washed with PBST to remove unbound plasmin. SAK or RGD-SAK in PBST containing 3% BSA was added into the wells at a final concentration of 200 nM for 2 h at the room temperature. After washing the plate with PBST for three times, unbound materials were removed. The SAK or RGD-SAK retained on the well was bound with polyclonal antibodies against SAK and HRP-conjugated anti-mouse secondary antibodies orderly. Substrate solution of TMB was added to develop the color and was stopped with 1 M H_2_SO_4_. The optical density was read at 450 nm for 30 min. As a blank, some wells were coated only with PBS but treated otherwise the same. The experiment was performed three times for both SAK and RGD-SAK.

### Fibrin clot lysis assay

Ninety-six-well microtiter plates were used for clot lysis assay according to the method reported previously [10]. The wells were loaded with thrombin (0.6 NIH unit/ml), human fibrinogen (1 mg/ml) and 20 mM CaCl_2 _in HBS buffer (10 mM HEPES, 130 mM NaCl, pH 7.4) and the polymerizing fibrin solution were incubated for 3 h at a room temperature. The clots formed were washed with HBS, and then layered with a solution containing 1.5 *μ*M hPlg and SAK, or RGD-SAK at varying concentrations (50, 100, 200 nM). After 30 min of incubation at room temperature, the surface of the clots was washed with HBS and the washed clots were layered with 1.5 *μ*M hPlg in HBS. The turbidity of the wells was measured as the optical density (OD) at 405 nm every 6 minutes, using a microtiter plate reader (Perkin Elmer 1420, VICTOR 3) until completion. *T*_50% _for clot lysis, which represented the time to achieve a 50% lysis of the fibrin clot, was calculated from the OD/time data. The concentration of fibrinolytic agent required to obtain 50% clot lysis in 3 hours (C_50_) was determined from plots of percent versus the concentration of test compound. Duplicate wells were prepared for each concentration of clot lysis of SAK or RGD-SAK in each experiment, and the experiment was repeated three times. As a control, some clots were layered only with HBS but treated otherwise the same.

### Measurement of platelet binding activity

An ELISA method was used to assess the platelet binding activity of SAK and RGD-SAK [26]. The activated human platelets induced by thrombin solution (0.2 IU/ml) were transferred to microtiter plate. SAK or RGD-SAK in the buffer A (50 mM Tris-HCl, 100 mM NaCl, 2 mM CaCl_2_, pH 7.4) was added to the wells at a final concentration of 25 nM, 50 nM, 100 nM, or 200 nM. After incubated in 37°C for 3 h, the plate was washed with PBST for three times to remove the unbound materials. SAK or RGD-SAK retained on the well was probed with polyclonal antibodies against SAK [27] followed by horseradish peroxidase (HRP)-conjugated anti-mouse secondary antibodies. The amount of HRP retained was assessed by using TMB (Tetrabenzidine) as HRP substrate. Color development was determined at 450 nm using a microplate reader (Perkin Elmer 1420, VICTOR 3). *T*_50% _for clot lysis, which represented the time to achieve a 50% lysis of the platelet-rich clot, was calculated from the OD/time data. The concentration of fibrinolytic agent required to obtain 50% clot lysis in 2 hours (C_50_) was determined from plots of percent versus the concentration of test compound. As a control, some wells were layered only with PBS but treated otherwise the same. The experiment was repeated three times.

### Platelet-rich clot lysis assay

Platelet-rich clot was formed using the modified procedure described by zhang *et al*. [26]. Clotting was initiated by mixing human thrombin (to 0.6 NIH unit/ml), human fibrinogen (1 mg/ml) and CaCl_2 _(to 20 mM) in human platelet. Each clot in the microtiter plate was layered with 100 *μ*l aliquot containing freshly mixed hPlg (1.5 *μ*M) and varied concentration of SAK or RGD-SAK (50, 100, 200 nM). After 30 min the surface of each clot was washed three times with HBS to remove unbound SAK or RGD-SAK and 100 *μ*l hPlg (1.5 *μ*M) were layered on each clot. The clot lysis process was monitored by measuring the turbidity using a microtiter plate reader. Duplicate wells were taken in each one of the three repeated experiments. As a control, some clots were layered only with HBS but treated otherwise the same.

### Inhibition of platelet aggregation

Platelet aggregation assay was performed with a modified method described by Seymour et al. [28]. Isolated Platelet-rich plasma (PRP) was incubated for 15 min at 37°C with either SAK (diluted by saline, 2.5, 5, 10, 20, 40 μM) or equimolar concentrations of RGD-SAK or RGD (Sigma, USA) prior to the platelet aggregation stimulation with ADP (10 μM) or collagen (1 μg/ml) or thrombin 1 U/ml. The aggregation response was recorded using a dual-channel aggregometer (APACT Labor, Germany) and aggregation results were expressed as percent inhibition of platelet aggregation. Saline was choosed to be control.

### Statistical analysis

Data are expressed as means ± SD. One-way analysis of variance (ANOVA) or student's *t *test was used where applicable. The differences with *P*-values < 0.05 were considered statistically significant.

## Authors' contributions

HSC and WM carried out most of the experimental work described in this paper and drafted the manuscript. YLZ assisted with the expression studies and data analysis. HBS carried out many molecular biology studies. HYS participated in design and coordination of the study and revised the manuscript in form and content. All authors read and approved the final manuscript.
